# Exome Sequencing of Extended Families with Alzheimer’s Disease Identifies Novel Genes Implicated in Cell Immunity and Neuronal Function

**DOI:** 10.4172/2161-0460.1000355

**Published:** 2017-07-31

**Authors:** HN Cukier, BK Kunkle, KL Hamilton, S Rolati, MA Kohli, PL Whitehead, J Jaworski, JM Vance, ML Cuccaro, RM Carney, JR Gilbert, LA Farrer, ER Martin, GW Beecham, JL Haines, MA Pericak-Vance

**Affiliations:** 1 John P. Hussman Institute for Human Genomics, University of Miami Miller School of Medicine, Miami, FL, USA; 2 Department of Neurology, University of Miami Miller School of Medicine, Miami, FL, USA; 3 John T. Macdonald Foundation, Department of Human Genetics, University of Miami Miller School of Medicine, Miami, FL, USA; 4 Mental Health and Behavioral Sciences Service, Miami Veterans Affairs, Miami, FL, USA; 5 Departments of Medicine, Neurology, Ophthalmology, Genetics and Genomics, Epidemiology and Biostatistics, Boston University, Boston, MA, USA; 6 Department of Population and Quantitative Health Sciences, Case Western Reserve University, Cleveland, OH, USA

**Keywords:** Alzheimer’s disease, dominant inheritance, linkage, multiplex, whole exome sequencing

## Abstract

**Objective:**

Alzheimer’s disease (AD) is a neurodegenerative disorder for which more than 20 genetic loci have been implicated to date. However, studies demonstrate not all genetic factors have been identified. Therefore, in this study we seek to identify additional rare variants and novel genes potentially contributing to AD.

**Methods:**

Whole exome sequencing was performed on 23 multi-generational families with an average of eight affected subjects. Exome sequencing was filtered for rare, nonsynonymous and loss-of-function variants. Alterations predicted to have a functional consequence and located within either a previously reported AD gene, a linkage peak (LOD>2), or clustering in the same gene across multiple families, were prioritized.

**Results:**

Rare variants were found in known AD risk genes including *AKAP9, CD33, CR1, EPHA1, INPP5D, NME8, PSEN1, SORL1, TREM2* and *UNC5C*. Three families had five variants of interest in linkage regions with LOD>2. Genes with segregating alterations in these peaks include *CD163L1* and *CLECL1*, two genes that have both been implicated in immunity, *CTNNA1*, which encodes a catenin in the cerebral cortex and *MIEF1*, a gene that may induce mitochondrial dysfunction and has the potential to damage neurons. Four genes were identified with alterations in more than one family include *PLEKHG5*, a gene that causes Charcot-Marie-Tooth disease and *THBS2*, which promotes synaptogenesis.

**Conclusion:**

Utilizing large families with a heavy burden of disease allowed for the identification of rare variants co-segregating with disease. Variants were identified in both known AD risk genes and in novel genes.

## Introduction

Alzheimer’s disease (AD) is the leading cause of dementia in the elderly [1]. The majority of individuals present with late-onset AD (≥ 65 years), but early-onset (<65 years) has also been reported in ~5% of cases. Both common genetic variants, such as the *APOE ε4* allele, and rare variants, have been found to impact the risk for both early- and late-onset AD [2–5]. While more than 20 genetic loci have been connected with late-onset AD to date, the underlying genetic architecture is complex and new risk genes are still being identified [6].

While genome-wide association studies (GWAS) have been key in identifying a majority of the novel regions of genetic risk in the past ten years, by design, GWAS are unlikely to recognize risk variants with rare frequencies in the population and necessitate the use of large cohorts of hundreds or even thousands of individuals to reach statistically significant conclusions [6]. In contrast, whole exome sequencing (WES) provides an alternative and complementary method to locate rare alterations in genes which may have medium to large effects on disease risk and require far fewer participants [6–8]. WES studies have identified new mutations in both known AD genes and novel risk genes, including *AKAP9, PLD3, TREM2* and *UNC5C*, as well as protective variants, such as those in *TREML2* [7–17]. Moreover, studying families with a heavy burden of AD and searching for genetic changes that segregate with disease can provide a unique opportunity to locate rare variants in novel risk genes such as *NOTCH3, PLD3* and *TTC3* [9,13,18]. These large AD families can reveal how multiple genetic variants may act in concert to influence risk [19–21]. For example, the *APOE ε2* allele was found to delay the age of onset by ~12 years in carriers of the E280A mutation in the *PSEN1* gene in the early-onset ‘Paisa’ pedigree [19]. In addition, genetic linkage can assist in narrowing genomic regions of interest potentially related to disease in large families [22]. In an effort to discover novel genes that may contribute to late-onset AD risk, we performed WES in 23 multiplex families that present with dominant inheritance patterns and prioritized variants that were inherited from common ancestors.

## Materials and Methods

### Patient ascertainment of extended AD families

240 individuals (77 AD subjects, 4 individuals with mild cognitive impairment (MCI) and 159 unaffected relatives) from 23 families of European ancestry heavily affected with late-onset AD were utilized in this study (Supplementary Table 1). All family members were recruited after providing informed consent and with approval by the relevant institutional review boards. Affected individuals meet the standard NINCDS-ADRDA criteria for AD and MCI [23–25]. In addition, cognitive and neuropsychiatric data were collected on all affected indivduals using the NCRAC LOAD battery, the Geriatric Depression Scale (GDS15), the Cornell Scale for Depression in Dementia (CSDD) and the Neuropsychiatric Inventory Questionnaire (NPIQ).

### Whole exome sequencing and variant detection

99 individuals (77 AD patients, 4 individuals with MCI, and 18 unaffected relatives) from 23 AD extended families underwent WES (Supplementary Table 1). Three micrograms of DNA from each sample were prepared using the SureSelect Human All Exon 50Mb Kit (Agilent Technologies) and the Paired-End Multiplexed Sequencing library kit (Illumina). Exome capture and sequence library construction was performed on a Sciclone G3 NGS Workstation (Caliper Life Sciences) and DNA was tested for uniform enrichment of targets with qPCR following established protocols provided by Agilent. Two exome sample libraries were sequenced per lane on a HiSeq 2000 Sequencing System (Illumina) in paired-end 2 × 100 bp runs. Sequencing data was processed using the Illumina RTA base calling pipeline v1.8. Reads were aligned to the human reference genome (hg19) with the Burrows- Wheeler Aligner (BWA) and variant calling performed with the Genome Analysis Toolkit (GATK) version 2.8 [26,27]. GATK parameters for variant quality control included duplicate sequence read removal, minimum read depth of 5, genotype quality (GQ) ≥ 20, variant quality score recalibration (VQSR, VQSLOD>0) and Genome Mappability Scores equal to 1 for the 35 base pair (bp) track and greater than or equal to 0.5 for the 20 bp track from the Duke Uniqueness Track [28]. The Duke uniqueness scores, generated for the ENCODE project and available as tracks in the University of California, Santa Cruz (UCSC) Genome Browser, report how unique a sequence is, where scores of 1 represent a completely unique sequence, a score of 0.5 indicates the sequence occurs exactly twice, and 0 represents the sequence occurs >4 times in the genome [29,30]. Small insertions and deletions were recognized by aligning the data with Bowtie2 and analyzing with the Pindel program [31,32].

### Genotyping and variant filtering

234 individuals, including all 99 samples that had WES, were evaluated by genome-wide SNP (single nucleotide polymorphism) arrays including the Human 1Mv1 BeadChip, the 1M-DuoV3 BeadChip, the HumanOmniExpress-12 v1.0 BeadChip, and the HumanOmni2.5-4v1 BeadChip. All chips were processed using the Tecan EVO-1 robot and BeadChips were scanned with either the Illumina BeadArray Reader or iScan. Data was extracted by the Genome Studio software and a GenCall cutoff score of 0.15 was used. Samples were required to have a genotyping call rate of 98% or higher, and SNPs a call rate of 95% or greater, to pass quality control. SNPs were only included in the analysis if they were present in at least 60% of samples across all platforms. Checks for relatedness, Mendelian inconsistencies, gender based on X-chromosome heterozygosity, and concordance between the genotypes of the variants identified through exome sequencing and genotyping were evaluated with PLINK version 1.07 [33]. All samples passed the quality control metrics.

Genotyping information was further used to delineate identical by descent (IBD) regions within each multiplex AD family. IBD filtering was implemented through the extended haplotype procedure in MERLIN version 1.1.2 [34]. Regions shared across all available AD individuals within a family were used to determine the IBD sharing segments and were, therefore, unique within each family. To determine the start and stop positions of IBD sharing regions within each family, the MERLIN output was evaluated in a sliding window of ten SNPs, defining IBD as sharing at each location with a threshold >50%.

### Linkage analysis

Nonparametric and parametric two-point and multipoint linkage analyses were performed using MERLIN [32]. A disease allele frequency of 0.0001 was used in an affecteds-only model for parametric analysis. PLINK was employed for LD pruning in the multipoint analysis, with CEU HapMap data as the reference population and the following settings: the indep-pairwise option with a window size of 50, a step of 5 and an r^2^ threshold of 0.5 [33,34].

### Variant annotation and prioritization

**Figure 1:**
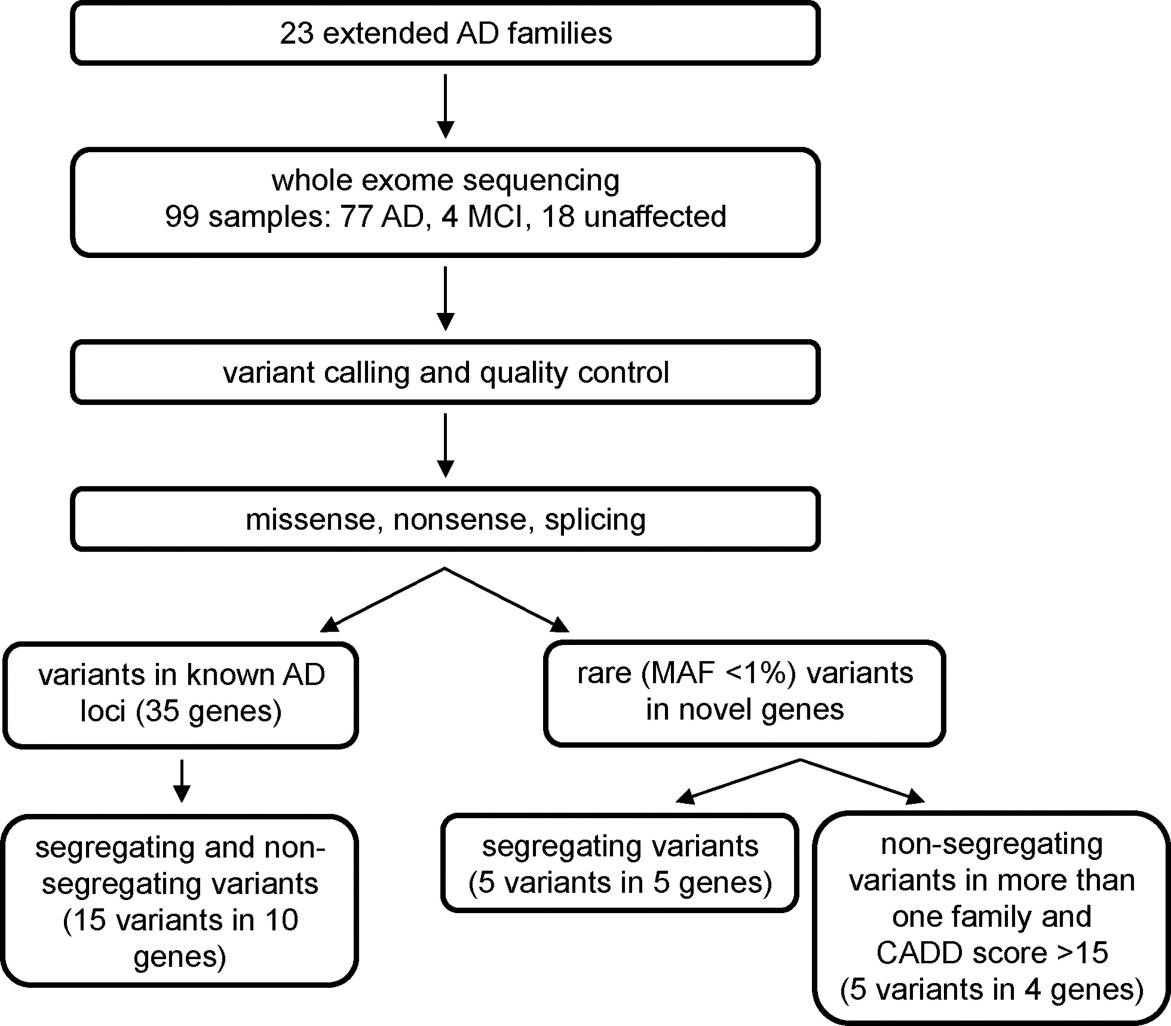
Study design. Strategy for processing the samples and prioritizing the variants that were resulting from whole exome sequencing.

Alterations passing quality measurements were annotated with the KGGSeq and ANNOVAR programs [35,36]. Variants were normalized prior to annotation [37]. Ensembl, RefSeq, and Gencode transcripts were all annotated, and the top consequence per gene was used for prioritization. CADD v1.3 scores were downloaded from the CADD server (http://cadd.gs.washington.edu/home) [38]. Figure 1 is an overview of the filtering and prioritization strategies used in this study. Brief descriptions of our three prioritization strategies are described below.

#### Variants in reported AD genes or loci

For all of the families, we evaluated whether variants were located in known AD risk genes; this includes genes identified in both early (*APP, PSEN1, PSEN2, GRN* and *TREM2*) and late-onset AD (Supplemental Table 2) [3,4]. Variants of interest were restricted to those with a minor allele frequency (MAF) ≤ 2% in the Kaviar Genomic Variant Database (version 160204-Public, 77,781 individuals) since these genes are known loci for AD [39]. The top variants of interest were validated by traditional Sanger sequencing.

#### Families with LOD scores >2

For each of the families, variants that segregated in all sequenced, affected individuals within areas LOD>2 were evaluated. Variants with a global MAF ≤ 1% in the Kaviar Database were prioritized. A MAF cutoff of ≤ 1% was implemented because variants with a MAF>1% in any ethnic population are unlikely to be a highly penetrant risk variant for AD [39–41]. This stricter MAF criteria was utilized to attempt to identify novel risk genes as opposed to variants in known AD genes. Variants were also prioritized based on their potential pathogenicity with the Combined Annotation-Dependent Depletion (CADD) score; scores ≥ 15 are predicted to be more likely to contribute to a disease risk as this score represents “the median value for all possible canonical splice site changes and non-synonymous variants” [38].

#### Variants and genes shared across families

Analysis across all 23 families was performed to identify if there were any genes with rare, nonsynonymous or loss-of-function (LOF) variants in more than one family. Variants were selected that had a MAF ≤ 1% in the Kaviar Database and CADD scores ≥ 15 to try to identify potentially damaging alterations [39].

### Association testing of top candidates

All top variants and genes from the three separate analyses described above were evaluated as potential risk variants using genome-wide association statistics for two family study cohorts (NIA-LOAD and MIRAGE) in the Alzheimer Disease Genetics Consortium [42,43]. Both gene and SNP-based tests were adjusted for age, sex and principal components (PCs). SNP-based logistic regression tests in each study were performed in the SNPTest program, and meta-analysis of these results was conducted using METAL [21,44]. Gene-based tests were conducted on meta-analysis summary statistics using VEGAS [44]. Variants tested in the gene-based analysis included all variants with a MAF<5%.

## Results

### Variants identified in known AD genes

Each sequenced family contained between 4 and 16 individuals diagnosed with AD. Mean age-at-onset across all families was 74.3 years. We identified 14 potentially damaging variants in 10 known AD genes and GWAS implicated loci (Table 1). Seven of the variants were observed in multiple affected individuals in the same family, while the remaining variants were observed only once. All alterations were single nucleotide changes with the exception of a four base pair deletion in *CD33*. This deletion is potentially the most deleterious as it is predicted to causes a frameshift that encodes two incorrect amino acids before terminating prematurely, thus failing to generate over 40% of the protein. In addition, multiple variants were observed in four genes: *AKAP9, INPP5D, SORL1* and *UNC5C*. Each gene had at least two families with a variant identified in it, while family 191 have a single affected individual with two alterations in *UNC5*. One of the variants in *UNC5C*, Ala860Thr, was identified in two different families; this alteration has a CADD score of 33, the highest score in this category.

### Segregating variants in linkage regions

Linkage scans aggregating all families identified one primary linkage region, a parametric multipoint peak on chromosome 1q23 (161.9–165.6 MB). Two families had strong linkage in this region (family specific LOD>2). However, no variants met our filtering criteria for these two families, suggesting the causal variant(s) may be non-coding changes either removed from by our filtering criteria or not present in our WES. Three of the 23 families also have family-specific parametric LOD scores >2; rare, potentially damaging alterations in five genes occurred within these regions and may potentially be the strongest novel AD candidate genes (Table 2). The five alterations were all missense changes in *CD163L1, CLECL1, CTNNA1, GALR3* and *MIEF1*.

### Genes with variants in more than one family

We identified four genes that had rare (MAF ≤ 1%), segregating, and potentially deleterious variants in at least two families (Table 3). Three of these genes had the same missense alteration identified in distinct families: *MKL2, PLEKHG5* and *THBS2*.

### Association testing of variants and genes

From our prioritized sets, a total of 9 SNPs and 14 genes were available for testing in the ADGC family-based meta-analysis datasets (NIA-LOAD and Mirage). None of the variants tested were significantly associated with disease (Supplemental Table 3). The gene *MIEF1*, identified as a candidate gene in a family 1201 with rare, potentially damaging segregating variants in a region with a LOD score of 2.22, reached nominal significance (p=0.049, Supplemental Table 4).

## Discussion

Through WES of large families with a heavy burden of AD, variants in both known and novel loci were identified that could contribute to risk. Filtering for rare, segregating, and potentially damaging variants identified five novel candidate genes (Table 2). These genes encompass a variety of functions that are suggestive of a link to AD. For example, two of these genes are involved in regulating immunity: *CD163L1* and *CLECL1* [45–47]. *CD163L1* is expressed in macrophages, upregulated in response to IL-10 and acts as an endocytic receptor [48]. *CLECL1* is highly expressed in B cells and dendritic cells and may enhance the immune response through upregulation of IL-4 [46]. Neuroinflammation has been shown to occur in AD patients, possibly through misregulation of microglia and triggered by amyloid beta plaques [49]. Additionally, established AD risk genes, such as *ABCA7, CD33* and *TREM2*, have also been linked to the immune system [4]. Another gene identified through this study, *CTNNA1*, encodes a catenin expressed at elevated levels in the nervous system [50]. *GALR3* is a receptor for the neuropeptide galanin, which has been shown to modulate a variety of processes, including cognition and memory, functions disrupted in AD [51,52]. *MIEF1* was nominally associated with late-onset AD in a meta-analysis of two family datasets from the ADGC, thereby suggesting that it may play a wider role in AD that extends beyond a single multiplex AD family. *MIEF1* may play a role in dysfunctional mitochondria and their potential to damage neurons [53,54]. Thus, each of the genes in the peak linkage regions are connected to known AD functions or neuronal pathways.

Four genes had rare, potentially damaging variants in more than one family (Table 3). When evaluating known functions of these genes, two are of particular interest, *PLEKHG5* and *THBS2*. *PLEKHG5* has been previously implicated in both Charcot-Marie-Tooth disease and spinal muscular atrophy [55,56]. *PLEKHG5* is ubiquitously expressed throughout the nervous system and murine studies demonstrated lowered expression can alter the velocity of nerve conduction [55,56]. In addition, *THBS2* is an intriguing novel AD candidate gene due to its involvement in synaptogenesis in immature astrocytes [57]. Further investigation into each of these novel AD candidates and the variants identified in this study is required.

After evaluating our families for rare alterations in known AD genes and loci, variants were discovered in genes previously connected to both early and late-onset AD (Table 1). Four genes had multiple alterations: *AKAP9, INPP5D, SORL1* and *UNC5C*. Some of these alterations have the potential to interfere with a protein’s function due to their location within specific domains. For example, a rare alteration in *UNC5C* identified in two distinct families, Ala860Thr, falls within the highly conserved DEATH domain, a region composed of alpha-helices and involved in apoptotic functions. Another study identified a different alteration in the same region in AD families and proposed that the alteration may increase the susceptibility of neurons to death [17]. A single affected individual in family 2349 was found to carry a frameshift deletion in *CD33* predicted to remove over 40% of the protein. There is evidence that higher expression of *CD33* in brains is associated with cognitive decline [58]. However, it may be that dysregulation of the protein, either through over or under expression, could contribute to AD risk. In addition, a rare alteration in *SORL1*, Thr588Ile, was identified within the vacuolar protein sorting 10 (VPS10) domain and may influence the processing of amyloid beta fragments, as has been shown for other AD associated variants in this gene [59]. Moreover, a potentially pathogenic alteration was identified in *PSEN1* in a single individual, Glu318Gly; this variant was previously reported to result in higher tau and phosphorylated tau levels in cerebrospinal fluids [60]. Three affected individuals from family 1893 were found to share the Arg336His alteration in the *NME8* gene, a change that fell within the first NDK domain of the protein. This gene has been associated with clinical features of AD including atrophy of the hippocampus and occipital gyrus [6,61]. These alterations, while not segregating within all affected individuals in the families, may play a contributing role in AD risk.

## Conclusion

This study demonstrates how using large, extended families to evaluate exome data identifies segregating risk variants in potentially novel AD candidate genes. In contrast to GWAS studies that have grown from hundreds to thousands and tens of thousands of participants, this study design requires far fewer participants. Indeed, a single extended family may be sufficient to identify a novel AD candidate gene [18]. Moreover, WES has the sensitivity to directly detect both common and rare variants that may confer a risk to AD, while GWAS findings are limited to pinpointing a region of interest, but not necessarily the causative alterations. In the study presented here, rare changes potentially contributing to AD risk were found in genes implicated in the immune response, *CD163L1* and *CLECL1*, and neuronal function, *CTNNA1, GALR3, MIEF1, PLEKHG5* and *THBS2*. Variants were also identified in genes previously connected to both early and late-onset AD including *AKAP9, INPP5D, SORL1* and *UNC5C*. Further investigation will be required to fully assess the cellular and molecular consequences of the alterations identified here as well as determine whether the novel genes found are involved in AD risk across larger datasets.

## Supplementary Material

Suppl tables

## Figures and Tables

**Table 1:** Known AD genes and loci with rare, potentially damaging variants.

Gene	Chr	Position (hg38)	Nucleotide	Amino acid	dbSNP	Kaviar MAF	CADD score	Family	Affected individuals with variant/ total affected sequenced
*AKAP9*	7	92002147	G>A	Glu756Lys	rs202091548	0.00008	27.8	191	1/2
*AKAP9*	7	92017092	G>A	Arg1288Gln	rs146797353	0.00822	6.2	419	2/2
*CD33*	19	51225851	CCCGG>C	Gly210Thrfs*2	rs201074739	0.01339	-	2349	1/2
*CR1*	1	207618089	A>G	Lys2308Arg	rs41274770	0.01463	11.9	1893	2/3
*EPHA1*	7	143398060	C>T	Arg492Gln	rs11768549	0.01214	17.39	701	1/3
*INPP5D*	2	233125865	G>A	Arg157Gln	rs200834931	0.00139	17.75	1399	2/2
*INPP5D*	2	233206711	C>A	Ala994Asp	rs187622749	0.00433	22.8	2349	2/2
*NME8*	7	37884315	G>A	Arg336His	rs62001869	0.01436	6.08	1893	3/3
*PSEN1*	14	73206470	A>G	Glu318Gly	rs17125721	0.01423	16.92	419	1/2
*SORL1*	11	121543625	C>T	Thr588Ile	rs752726649	0.00001	32	191	2/2
*SORL1*	11	121627591	C>T	Thr2134Met	rs142884576	0.00023	28.6	1240	1/2
*TREM2*	6	41161469	C>T	Arg92His	rs143332484	0.00791	11.11	1893	1/3
*UNC5C*	4	95170263	C>T	Ala860Thr	rs34585936	0.01808	33	191	2/2
*UNC5C*	4	95170263	C>T	Ala860Thr	rs34585936	0.01808	33	2119	1/2
*UNC5C*	4	95202928	G>A	Pro666Ser	rs760453427	0.00001	20.2	191	1/2

**Table 2:** Families with segregating, rare, potentially damaging variants in high LOD regions.

Family	Affected individuals with variant	LOD score	Gene	Chr	Position (hg38)	Nucleotide	Amino acid	dbSNP	Kaviar score	CADD score
757	9	2.95	*CD163L1*	12	7369477	T>C	Thr1317Ala	rs150384982	0.00137	3.73
757	9	2.95	*CLECL1*	12	9722727	T>C	Thr135Ala	rs118152239	0.00769	0.03
911	7	2.36	*CTNNA1*	5	138824559	G>C	Gln206His	rs150893072	0.0043	23.2
1201	5	2.22	*GALR3*	22	37823563	C>G	Pro53Ala	rs78650836	0.00328	10.31
1201	5	2.22	*MIEF1*	22	39512414	C>T	Arg169Trp	rs2232088	0.00525	34

**Table 3:** Genes with rare, potentially damaging variants in more than one family.

Gene	Family	Chr	Position (hg38)	Nucleotide	Amino acid	dbSNP	Kaviar score	CADD score	Affected individuals with variant/ total affecteds sequenced
*DAAM2*	191	6	39861001	A>G	Tyr128Cys	rs201047462	0.00017	28.00	2/2
	716	6	39884013	C>T	Arg680Trp	rs200964833	0.00019	35.00	3/3
*MKL2*	1893	16	14245640	T>C	Ser398Pro	rs113935526	0.00479	19.20	3/3
	26044	16	14245640	T>C	Ser398Pro	rs113935526	0.00479	19.20	2/3
*PLEKHG5*	803	1	6496525	G>A	Pro40Ser	rs201669114	0.00148	27.00	2/2
	1008	1	6496525	G>A	Pro40Ser	rs201669114	0.00148	27.00	2/2
*THBS2*	1240	6	169220312	C>A	Val1133Phe	rs112533700	0.00017	29.50	2/2
	1893	6	169220312	C>A	Val1133Phe	rs112533700	0.00017	29.50	3/3
