# Supplementary material for: Exome Sequencing of Extended Families with Alzheimer’s Disease Identifies Novel Genes Implicated in Cell Immunity and Neuronal Function
Source: J Alzheimers Dis Parkinsonism. Author manuscript; Available in PMC 2017 Nov 22. (PMC5698805; doi:10.4172/2161-0460.1000355)
Supplement: Suppl tables [file NIHMS907692-supplement-Suppl_tables.pdf]

Supplementary Data

Exome sequencing of extended families with Alzheimer's disease identifies novel genes implicated in cell immunity and neuronal function

Holly N. Cukier, PhD[1,2]*, Brian W. Kunkle, PhD, MPH[1]*, Kara L. Hamilton-Nelson, MPH[1], Sophie Rolati, MS[1], Martin A. Kohli, PhD[1], Patrice L. Whitehead, BS[1], James M. Jaworski, BS[1],Jeffery M. Vance, MD, PhD[1,3], Michael Cuccaro, PhD[1,3], Regina M. Carney, MD[1,4], John R. Gilbert, PhD[1,3], Lindsay A. Farrer, PhD[5], Eden R. Martin, PhD[1,3], Gary W. Beecham, PhD[1,3], Jonathan L. Haines, PhD[56], Margaret A. Pericak-Vance, PhD[1,2,3]◆

[1]John P. Hussman Institute for Human Genomics, University of Miami Miller School of Medicine, Miami, FL, USA
[2]Department of Neurology, University of Miami Miller School of Medicine, Miami, FL, USA
[3]Dr. John T. Macdonald Foundation Department of Human Genetics, University of Miami Miller School of Medicine, Miami, FL, USA
[4]Mental Health and Behavioral Sciences Service, Miami Veterans Affairs, Miami, FL, USA
[5]Departments of Medicine, Neurology, Ophthalmology, Genetics & Genomics, Epidemiology, and Biostatistics, Boston University, Boston, MA, USA
[6]Department of Population & Quantitative Health Sciences, Case Western Reserve University, Cleveland, OH, USA

*Holly N. Cukier and Brian W. Kunkle should be considered joint first authors.

The authors have no conflict of interest to declare.

◆Corresponding author:
Margaret A. Pericak-Vance, Ph.D.
Director, John P. Hussman Institute for Human Genomics
Executive Vice Chair, Dr. John T Macdonald Foundation Department of Human Genetics
University of Miami Miller School of Medicine
1501 NW 10th Avenue, BRB-314 (M860)
University of Miami, Miller School of Medicine, Miami, Florida 33136
Tel: (305) 243-2308
Fax: (305) 243-2396
mpericak@med.miami.edu

Supplementary Tables

Supplementary Table 1. LOAD Families

family	diagnosed AD & MCI family members	Individuals Exome Sequenced		
		AD cases	MCI cases	unaffected relatives
191	5	2	0	0
419	6	2	0	1
609	7	2	0	1
701	9	3	0	0
713	9	6	0	1
716	6	3	0	1
757	14	10	1	1
803	6	2	1	0
911	11	7	0	1
974	11	7	2	0
1008	4	2	0	1
1086	9	4	0	3
1201	16	5	0	0
1240	6	2	0	1
1399	9	2	0	1
1743	7	2	0	1
1893	5	3	0	0
2119	6	2	0	0
2120	5	2	0	1
2349	5	2	0	1
26044	7	3	0	1
26284	6	2	0	1
33035	7	2	0	1
	176	77	4	18

Supplemetary Table 2. Known AD genes and loci

gene	chr	reference
ABCA7	19	Lambert, et al, Nat Genet, 2013
AKAP9	7	Logue, et al, Alzheimers Dement, 2014
APOE	19	Corder, et al, Science, 1993
APP	21	Goate, et al, Nature, 1991
BIN1	2	Lambert, et al, Nat Genet, 2013
CASS4	20	Lambert, et al, Nat Genet, 2013
CD2AP	6	Lambert, et al, Nat Genet, 2013
CD33	19	Lambert, et al, Nat Genet, 2013
CELF1	11	Lambert, et al, Nat Genet, 2013
CLU	8	Lambert, et al, Nat Genet, 2013
CR1	1	Brouwers, et al, Mol Psychiatry, 2012
EPHA1	7	Lambert, et al, Nat Genet, 2013
FERMT2	14	Lambert, et al, Nat Genet, 2013
INPP5D	2	Lambert, et al, Nat Genet, 2013
MADD	11	Lambert, et al, Nat Genet, 2013
MAPT	17	Jun, et al, Molec Psychiatry, 2015
MEF2C	5	Lambert, et al, Nat Genet, 2013
MS4A2	11	Lambert, et al, Nat Genet, 2013
MS4A3	11	Lambert, et al, Nat Genet, 2013
MS4A4A	11	Lambert, et al, Nat Genet, 2013
MS4A4E	11	Lambert, et al, Nat Genet, 2013
MS4A6A	11	Lambert, et al, Nat Genet, 2013
MS4A6E	11	Lambert, et al, Nat Genet, 2013
NME8	7	Lambert, et al, Nat Genet, 2013
NYAP1	7	Lambert, et al, Nat Genet, 2013
PICALM	11	Lambert, et al, Nat Genet, 2013
PSEN1	14	Sherrington, et al, Nature, 1995
PSEN2	1	Levy-Lahad, et al, Science, 1995
PTK2B	8	Lambert, et al, Nat Genet, 2013
RIN3	14	Lambert, et al, Nat Genet, 2013
SLC24A4	14	Lambert, et al, Nat Genet, 2013
SORL1	11	Rogaeva, et al, Nat Genet, 2007
TREM2	6	Lambert, et al, Nat Genet, 2013
UNC5C	4	Wetzel-Smith, et al, Nat Med, 2014
ZCWPW1	7	Lambert, et al, Nat Genet, 2013

Supplementary Table 3. SNPs tested in the ADGC

dbSNP	MAF	effect	StdErr	p-value	HetPVal
rs150893072	0.0103	0.0508	0.2508	0.8396	0.6873
rs143332484	0.007	0.0797	0.3541	0.8219	0.4185
rs17125721	0.026	-0.0224	0.1353	0.8684	0.0826
rs41274770	0.021	-0.0971	0.2098	0.6436	0.0113
rs118152239	0.0126	-0.0193	0.1568	0.9020	0.5985
rs34585936	0.0219	0.2669	0.1735	0.1240	1.0000
rs62001869	0.0129	0.1814	0.194	0.3497	0.0216
rs11768549	0.0153	0.0388	0.1884	0.8369	0.8544
rs142692233	0.0563	-0.1794	0.1169	0.1250	0.3298
rs2232088	0.0158	0.1606	0.1997	0.4213	0.0020

Supplementary Table 4. Genes tested in the ADGC

gene	chr	start	end	SNPs	p-value
CD163L1	12	7499281	7632493	169	0.6139
CD33	19	51728320	51747115	64	0.7228
CLECL1	12	9868456	9885895	40	0.6040
CR1	1	207669492	207813992	134	0.9010
CTNNA1	5	137946656	138270723	284	0.0990
DAAM2	6	39760142	39872648	193	0.1782
EPHA1	7	143087382	143105985	71	0.2970
MIEF1	22	39895437	39914137	57	0.0495
MKL2	16	14165178	14360630	203	0.5149
NME8	7	37888199	37940003	106	0.8911
PLEKHG5	1	6526152	6580121	95	0.1485
PSEN1	14	73603126	73690399	119	0.2376
SORL1	11	121322912	121504402	231	0.1089
